# Role of Posterior Occlusal Support in the Development of Frailty Among Japanese Adults: A Longitudinal Cohort Study

**DOI:** 10.1111/ggi.70405

**Published:** 2026-02-08

**Authors:** Takashi Miyano, Taro Kusama, Yudai Tamada, Ken Osaka, Kenji Takeuchi

**Affiliations:** ^1^ Department of Medical and Robotic Engineering Design Tokyo University of Science Tokyo Japan; ^2^ Department of International and Community Oral Health Tohoku University Graduate School of Dentistry Sendai Japan; ^3^ Division of Statistics and Data Science, Liaison Center for Innovative Dentistry Tohoku University Graduate School of Dentistry Sendai Japan

**Keywords:** claim data, Eichner classification, frailty, longitudinal study, occlusal contacts

## Abstract

**Aim:**

Recent studies have suggested that masticatory function may influence frailty progression. However, the relationship between posterior occlusal support and frailty remains underexplored. We aimed to evaluate the association between the loss of posterior occlusal support and frailty in a large cohort of Japanese adults.

**Methods:**

This retrospective cohort study used data from the JMDC Claims Database. We included 386 270 individuals aged ≥ 40 years who underwent both specific health checkups and dental visits in 2016, with follow‐up data available through 2020. We categorized posterior occlusal support using the Eichner classification (A, all support zones intact; B, partial support zones; C, no functional support zones). We assessed frailty annually using the claims‐based frailty index (CFI) (a deficit accumulation‐type frailty measure). We used generalized estimating equations to evaluate the association between occlusal support and frailty after adjusting for potential confounders.

**Results:**

At baseline, the mean age was 49.6 (standard deviation, 7.2) years, and 47.4% of the participants were women. The prevalence of frailty was 4.9%, 9.5%, and 12.1% in the Eichner A, B, and C groups, respectively. Compared to Eichner A, the adjusted odds ratios (ORs) for frailty were 1.21 (95% confidence interval [CI], 1.13–1.30) for Eichner B and 1.39 (95% CI, 1.21–1.59) for Eichner C. This relationship was stronger among women than men.

**Conclusions:**

Reduced posterior occlusal support is independently associated with an increased risk of frailty. These findings underscore the importance of oral health in preventing physical debilitation and suggest incorporating early dental interventions into health strategies for older populations.

## Graphical abstract

Loss of posterior occlusal support was independently associated with an increased risk of frailty in a nationwide longitudinal analysis, highlighting the systemic importance of maintaining functional occlusion to promote healthy aging.

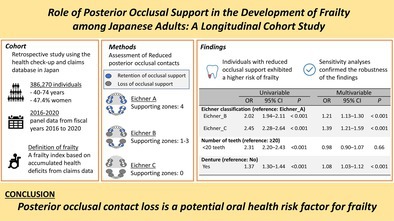

## 1 Introduction

Frailty is an emerging global public health challenge in aging societies that increases the risk of disability, falls, hospitalization, and mortality [1]. Systematic reviews and meta‐analyses have estimated that 10%–24% of community‐dwelling older adults exhibit frailty, highlighting its widespread prevalence and clinical significance [2, 3]. Unlike many age‐related conditions, frailty is a dynamic state that can be mitigated or reversed through timely intervention. This underscores the urgent need to identify the modifiable risk factors that contribute to its development.

Recently, oral health has gained attention as a potential determinant of frailty. Longitudinal studies have also suggested that tooth loss is associated with frailty [4, 5]. However, the number of teeth alone does not fully capture masticatory function [6]. Efficient mastication plays a critical role in maintaining nutritional intake and overall well‐being, as impaired chewing ability can limit food choices, reduce dietary quality, and diminish the pleasure of eating [7]. Notably, recent evidence indicates that masticatory function may be more strongly linked to frailty than tooth count [8]. However, the findings remain inconsistent; while some studies support this association [9], others report no significant relationship [10]. A possible explanation for these discrepancies is that masticatory function is influenced by multiple factors beyond the tooth count, including occlusion status, position of the remaining teeth, and occlusal force. Among these, posterior occlusal support plays a particularly crucial role as it is strongly correlated with masticatory performance [11]. Given this relationship, it is plausible that the loss of posterior occlusal support accelerates frailty progression.

Frailty is typically assessed using two validated approaches: the frailty phenotype, which focuses on physical criteria such as weight loss and weakness; and the deficit accumulation frailty index, which quantifies frailty based on accumulated health deficits [12, 13]. While previous studies have examined the association between masticatory ability and frailty, most have relied on phenotypic models and were constrained by limited sample sizes (typically ranging from a few hundred to a few thousand individuals), short follow‐up durations, and an insufficient capacity to capture temporal dynamics in health trajectories [14, 15, 16, 17, 18, 19]. These methodological limitations hinder the generalizability of prior findings and underscore the need for large‐scale longitudinal analyses capable of capturing temporal relationships in frailty progression. Japan's universal health insurance system offers a unique opportunity to address these challenges by integrating nationwide administrative claims data. Recently, a claims‐based frailty index (CFI) modeled using the deficit accumulation approach was validated for use with Japanese claims records [20]. This tool facilitates comprehensive and scalable frailty assessments across large populations, enabling epidemiological studies that are both statistically robust and reflective of real‐world clinical contexts.

In this study, we hypothesized that the loss of posterior occlusal support would significantly affect the progression of frailty. To verify this hypothesis, we used a nationwide longitudinal dataset to investigate the association between posterior occlusal support, as classified using the Eichner classification, and frailty progression based on the CFI.

## 2 Methods

### 2.1 Study Design and Participants

We used the JMDC Claims Database, a nationwide insurer‐based database in Japan that integrates administrative medical and dental claims and health checkup data collected from over 60 health insurance associations, primarily for employees and their dependents aged < 75 years.

**FIGURE 1:**
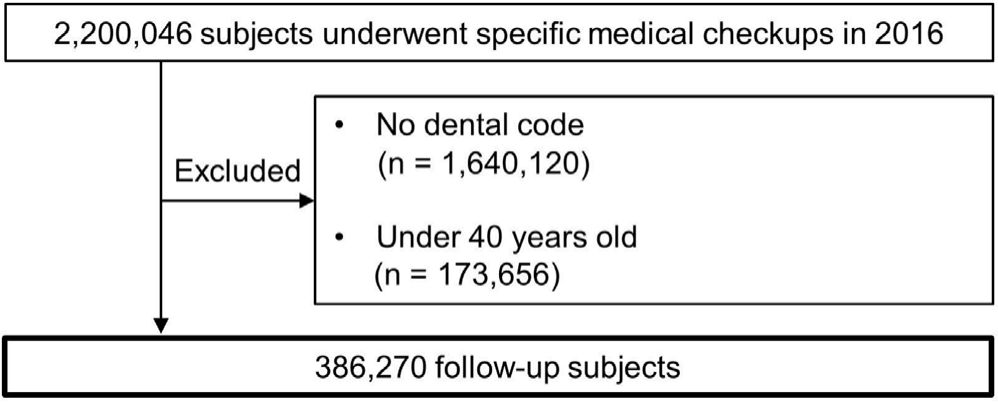
Flow diagram of participant selection. This flowchart outlines the sequential process of selecting study participants from the JMDC Claims Database. The initial dataset included 2 200 046 individuals who underwent health checkups in 2016. The exclusion criteria were as follows: Individuals who did not have dental codes for all 28 teeth within 1 year before the health check‐up (*n* = 1 640 120) and those who were aged < 40 years (*n* = 173 656).

We constructed panel data using five time‐points from fiscal years 2017 to 2020, with 2016 as the baseline year. Figure 1 illustrates the participant selection process. From an initial cohort of 2 200 046 individuals who underwent specific health examinations in 2016, we excluded those without dental claims (*n* = 1 640 120) and those aged < 40 years (*n* = 173 656), resulting in an analytical sample of 386 270 participants.

### 2.2 CFI Assessment

We assessed CFI using the methodology developed by Nakatsuka et al., leveraging medical claims data to quantify frailty in Japan [20]. Briefly, CFI was calculated based on the International Classification of Diseases, 10th Revision (ICD‐10), excluding ICD‐10 codes flagged as uncertain diagnoses. We categorized certain ICD‐10 codes into 52 distinct groups and recorded the presence of any diagnosis within each group over the past year. We assigned each of the 52 groups a specific weight, and the aggregated score was used as the CFI score (Table S1). The CFI ranged from 0 to 1, with higher scores indicating greater frailty; a score of ≥ 0.25 was classified as “frail.”

### 2.3 Eichner Classification

The Eichner classification was determined based on dental claims data. This study identified the number of remaining teeth and their positions using dental formulas, indicating the presence and condition of each tooth [21, 22]. To assess posterior occlusal support, we recorded the number of supporting zones—defined as pairs of opposing premolars and molars in occlusal contact on both the right and left sides—and categorized them into four levels (0–4 support zones) [23]. Based on this, we applied the Eichner classification to categorize occlusal conditions into three groups: Class A, occlusal contact in all four posterior support zones; Class B, occlusal contact in one to three posterior zones or only in the anterior region; and Class C, absence of occlusal contact.

### 2.4 Other Variables

We adjusted for demographic characteristics, comorbidities, and health behaviors that are commonly associated with frailty [24] and could be extracted from the JMDC database. The demographic variables included sex and age at health checkup (40–59 or 60–74 years). We assessed comorbidities using the Charlson Comorbidity Index (CCI) (CCI score, 0 or ≥ 1) and diagnoses of depression (ICD‐10 codes, F32 and F33) and dementia (ICD‐10 codes, F00–F03 and G30–G31, excluding G319) [25]. The CCI assigns weighted scores (1–6) to various chronic conditions, with the total score reflecting the overall disease burden [26]. Health behaviors included body mass index (BMI), smoking, alcohol consumption, and physical activity. Participants were classified by BMI as underweight (< 18.5), normal (18.5–24.9), or obese (≥ 25.0). Smoking status was self‐reported, with current smokers defined as those who had smoked ≥ 100 cigarettes and continued smoking within the past 28 days. Alcohol consumption was categorized by frequency, identifying current drinkers as those who consumed alcohol regularly or occasionally. Physical activity was assessed using a standardized questionnaire, with adequate activity defined as exercising ≥ 30 min twice a week or walking ≥ 1 h per day. We categorized the number of remaining teeth (excluding third molars) into two groups: < 20 or ≥ 20 teeth, indicating that having ≥ 20 teeth is generally sufficient to maintain functional mastication [27]. Denture use was determined based on the presence of claims for denture management fees (procedure codes: 308002510, 308002610, 308002710, or 308004210) recorded during the period used to assess the number of remaining teeth [21]. Table S2 presents the definitions of the variables used in this study.

### 2.5 Statistics

We compared the baseline characteristics of the participants before and after missing value imputation. We performed multiple imputations to minimize the selection bias associated with missing values. We employed a data augmentation technique based on a multivariate normal distribution to generate 10 imputed datasets. The estimates from these datasets were combined using Rubin's rule [28]. We used longitudinal data from the CFI, collected over five time‐points from 2016 to 2020, to construct patient‐level panel data, enabling the assessment of frailty progression over time in relation to the status of posterior occlusal contact at baseline. The time‐variant variables were CFI and time‐point, and the time‐invariant variables were all covariates. We used a generalized estimating equation (GEE) model, which extends generalized linear models to account for correlated measurements over time and estimates population‐level changes, to evaluate the association between the Eichner classification and frailty trajectories over time. Both unadjusted and adjusted models were constructed, with the latter accounting for the number of teeth, denture use, demographic characteristics, comorbidities, health behaviors, and time‐points. To assess differences in frailty trajectories between groups, the adjusted models included a time interaction term (Eichner classification × time‐points). We estimated odds ratios (ORs) and corresponding 95% confidence intervals (CIs) using logistic regression with GEEs. To test the robustness of our results, we conducted four sensitivity analyses: using complete cases only (*n* = 328 873), applying continuous rather than categorical variables (age group, obesity, thinness, and tooth group) in the multivariable model, performing sex‐stratified analyses (230 308 men and 155 962 women), and calculating E‐values to assess the potential impact of unmeasured confounding. All analyses were performed using R Studio, version 4.0.4 (R Foundation for Statistical Computing, Vienna, Austria).

## 3 Results

This study analyzed data from 386 270 participants (average age, 49.6 ± 7.2 years), of whom 47.4% were women. Table 1 shows the baseline characteristics of the study population. Table 2 summarizes the prevalence of frailty at different time‐points, categorized according to the Eichner classification. In 2016, the frailty rates were 4.9%, 9.5%, and 12.1% for the Eichner A, B, and C groups, respectively, indicating a progressive increase over time.

**TABLE 1:** Characteristics of the participants.

Baseline characteristics	Original data		Imputed data sets	
	*N*	%	*N*	%
Eichner classification, *N* (%)				
Eichner A	372 506	96.4	372 506	96.4
Eichner B	10 966	2.9	10 966	2.9
Eichner C	2798	0.7	2798	0.7
Age group, *N* (%)				
40–59 years	343 766	89.0	343 766	89.0
60–75 years	42 504	11.0	42 504	11.0
Sex, *N* (%)				
Men	230 308	52.6	230 308	52.6
Women	155 962	47.4	155 962	47.4
BMI, *N* (%)				
Obese	91 441	23.7	91 741	23.8
Normal	264 509	68.5	265 126	68.6
Underweight	29 332	7.6	29 403	7.6
Missing	988	0.2	—	—
CCI, *N* (%)				
0	311 007	80.5	311 007	80.5
≥ 1	75 263	19.5	75 263	19.5
Depression, *N* (%)				
No	311 007	80.5	311 007	80.5
Yes	75 263	19.5	75 263	19.5
Dementia, *N* (%)				
No	386 145	100.0	386 145	100.0
Yes	125	0.0	125	0.0
Physical inactivity, *N* (%)				
No	120 981	31.3	139 511	36.1
Yes	213 192	55.2	246 759	63.9
Missing	52 097	13.5	—	—
Current smoking, *N* (%)				
No	299 709	77.6	308 791	79.9
Yes	75 178	19.5	77 479	20.1
Missing	11 383	2.9	—	—
Alcohol, *N* (%)				
No	89 794	23.2	98 108	25.4
Yes	264 085	68.4	288 162	74.6
Missing	32 391	8.4	—	—
Number of teeth, *N* (%)				
≥ 20 teeth	379 948	98.4	379 948	98.4
1–19 teeth	6322	1.6	6322	1.6
Denture use, *N* (%)				
No	376 979	97.6	376 979	97.6
Yes	9291	2.4	9291	2.4

*Note:* Eichner's classification was applied based on the number of posterior support zones (0–4), defined as pairs of opposing premolars and molars in occlusal contact. Class A: occlusal contact in all four posterior support zones; Class B: occlusal contact in one to three posterior support zones or only in the anterior region; Class C: absence of occlusal contact.

Abbreviations: BMI, body mass index; CCI, Charlson Comorbidity Index.

**TABLE 2:** Cross‐tabulation of the onset of frailty by Eichner classification in survey waves.

	Frailty (*N*, %)				
	2016 (Baseline)	2017	2018	2019	2020
Eichner A (*N* = 372 506)	18 256 (4.9)	21 768 (5.8)	24 009 (6.4)	25 787 (6.9)	23 898 (6.4)
Eichner B (*N* = 10 966)	1040 (9.5)	1293 (11.8)	1388 (12.0)	1408 (12.8)	1316 (12.0)
Eichner C (*N* = 2798)	339 (12.1)	393 (14.0)	444 (13.9)	422 (15.1)	389 (13.9)

*Note:* Eichner's classification was applied based on the number of posterior support zones (0–4), defined as pairs of opposing premolars and molars in occlusal contact. Class A: occlusal contact in all four posterior support zones; Class B: occlusal contact in one to three posterior support zones or only in the anterior region; Class C: absence of occlusal contact.

GEEs were used to examine the association between the Eichner classification and frailty (Table 3). Even after controlling for demographic characteristics, comorbid conditions, health‐related behaviors, and tooth count, the Eichner classification was significantly associated with frailty. Compared to the Eichner A group, the odds of frailty were 1.21 times higher in the Eichner B group (OR, 1.21; 95% CI, 1.13–1.30; *p* < 0.001) and 1.39 times higher in the Eichner C group (OR, 1.39; 95% CI, 1.21–1.59; *p* < 0.001). An interaction term between Eichner classification and time‐points showed no significant interaction effects (Table S3).

**TABLE 3:** Univariable and multivariate GEE model for frailty.

	Univariable			Multivariable		
	OR	95% CI	*p*	OR	95% CI	*p*
Eichner classification (reference: Eichner_A)						
Eichner_B	2.02	1.94–2.11	< 0.001	1.21	1.13–1.30	< 0.001
Eichner_C	2.45	2.28–2.64	< 0.001	1.39	1.21–1.59	< 0.001
Number of teeth (reference: ≥ 20)						
< 20 teeth	2.31	2.20–2.43	< 0.001	0.98	0.90–1.07	0.66
Denture (reference: No)						
Yes	1.37	1.30–1.44	< 0.001	1.08	1.03–1.12	< 0.001
Age group (reference: 29–59 years)						
60–75 years	2.66	2.60–2.72	< 0.001	3.69	3.60–3.79	< 0.001
Sex (reference: Male)						
Female	0.99	0.97–1.01	0.20	1.12	1.10–1.14	< 0.001
BMI (reference: Normal)						
Underweight	0.91	0.88–0.94	< 0.001	1.04	1.00–1.08	0.06
Obese	1.64	1.60–1.67	< 0.001	1.69	1.66–1.73	< 0.001
Smoking (reference: No)						
Yes	0.89	0.87–0.91	< 0.001	0.96	0.94–0.98	0.001
Alcohol (reference: No)						
Yes	1.06	1.04–1.08	< 0.001	1.12	1.10–1.15	< 0.001
CCI (reference: 0)						
≥ 1	1.41	1.38–1.44	< 0.001	1.26	1.23–1.29	< 0.001
Depression (reference: No)						
Yes	5.09	4.87–5.33	< 0.001	5.48	5.23–5.74	< 0.001
Dementia (reference: No)						
Yes	13.24	10.7–16.4	< 0.001	7.71	5.87–10.1	< 0.001
Physical activity (reference: Yes)						
No	1.01	0.99–1.03	0.35	1.06	1.04–1.08	< 0.001
Year						
1‐year increase	1.07	1.07–1.08	< 0.001	1.07	1.07–1.08	< 0.001

*Note:* Eichner's classification was applied based on the number of posterior support zones (0–4), defined as pairs of opposing premolars and molars in occlusal contact. Class A: occlusal contact in all four posterior support zones; Class B: occlusal contact in one to three posterior support zones or only in the anterior region; Class C: absence of occlusal contact. The GEE model included frailty as the outcome and the Eichner classification as the explanatory variable.

Abbreviations: BMI, body mass index; CCI, Charlson comorbidity index; CI, confidence interval; GEE, generalized estimating equation; OR, odds ratio.

Robustness was confirmed using four sensitivity analyses. First, to examine the potential impact of missing data, we conducted a complete‐case analysis that excluded imputed data. The results remained consistent with those of the primary analysis (Table S4), suggesting that the missing data did not substantially bias the findings. Second, replacing the categorical variables with continuous measures of age, BMI, and tooth count produced similar OR estimates (Table S5). Third, because a significant interaction between the Eichner classification and sex was observed (Table S6), we performed sex‐stratified analyses. The association between occlusal contact loss and frailty was more pronounced in women, with ORs of 1.28 (95% CI, 1.14–1.44) for Eichner B and 1.57 (95% CI, 1.24–1.99) for Eichner C (Table S7). Lastly, an E‐value analysis suggested that an unmeasured confounder would need a minimum association strength of 1.71 (lower confidence limit, 1.51) for Eichner B and 2.13 (lower confidence limit, 1.71) for Eichner C with both the exposure and outcome to fully explain the observed associations (Table S8). These findings were consistent across multiple analytical approaches, supporting the reliability of the observed association between occlusal support loss and frailty.

## 4 Discussion

This retrospective observational study investigated the relationship between posterior occlusal contact and the risk of frailty progression. Our findings indicate that the Eichner B and C groups, characterized by reduced or absent posterior occlusal support, had a significantly higher risk of developing frailty than the Eichner A group. This association remained robust across multiple sensitivity analyses, reinforcing the potential role of diminished occlusal function as an independent risk factor for frailty.

A key strength of this study lies in its use of the CFI, derived from Japanese claims data, to objectively assess frailty in a large‐scale population. By leveraging this extensive dataset, we elucidated a novel association between posterior occlusal contact and frailty, offering insights into how dental health influences systemic aging processes. These findings indicate an association between reduced posterior occlusal support and a higher risk of frailty. Posterior occlusal support may serve as a potential focus for future interventional research to explore whether maintaining or restoring occlusal function could influence frailty progression.

Several studies involving older adults have demonstrated that reduced masticatory ability is associated with frailty. Measures of masticatory function included maximum bite force, mixing ability, and self‐reported masticatory ability. Frailty was assessed using criteria such as those of Fried, Cardiovascular Health Study, or the Kihon Checklist (Japanese Ministry of Health). Cross‐sectional reports have revealed statistically significant correlations between masticatory measures and frailty in older Japanese adults [29, 30]. Longitudinal studies (2–5 years) have shown that poor masticatory performance precedes frailty [15, 17, 31]. A 5‐year study reported that individuals in the lowest maximum bite force tertile had a 2.78‐fold higher risk of frailty [15], and another study found a 2.4‐fold increased risk of physical frailty linked to impaired masticatory ability [31]. Notably, the assessment of loss of posterior occlusal support using the Eichner classification also confirmed its effect on the progression of frailty, similar to other assessments of masticatory ability. A previous longitudinal study that evaluated both the maximum bite force and Eichner classification demonstrated that each was significantly associated with the risk of frailty in univariate analyses; however, when both variables were included in a multivariate model, the association between the Eichner classification and frailty was no longer statistically significant, while maximum bite force remained a robust predictor [15]. In that study, 62.4% of the denture‐wearers underwent bite force measurements while wearing their dentures, suggesting that occlusal function may have been partially compensated. In contrast, the present study evaluated posterior occlusal support based exclusively on the condition of the natural dentition, allowing for a more direct and unconfounded assessment of the association between natural occlusion and frailty risk, independent of prosthetic compensation. Notably, we found a stronger association in women, aligning with the sex differences in oral health and frailty trajectories observed in other studies [3].

Although the precise mechanisms linking the loss of posterior occlusal support to frailty remain unclear, its impact on cognitive function, motor ability, and nutritional intake is likely crucial. Reduced masticatory function due to diminished occlusal support decreases the afferent stimulation of brain regions involved in cognitive processing, thereby increasing the risk of cognitive impairment and dementia [25, 32]. Functional imaging studies have demonstrated that mastication enhances blood oxygenation level‐dependent signals in the sensorimotor cortex, supplementary motor area, insular cortex, thalamus, and cerebellum. This suggests that occlusal conditions influence neural activity through changes in cerebral blood perfusion, potentially supporting cognitive function [33, 34]. In addition to cognitive effects, impaired occlusion is associated with a slower walking speed, which is a well‐established indicator of physical frailty [35]. Studies have also identified a link between occlusal force and one‐leg standing time, which is a recognized measure of balance and postural control. [36] A proposed mechanism underlying this association is the loss of periodontal ligament mechanoreceptors, which provide essential proprioceptive input to the central nervous system. Degeneration of these receptors may contribute to postural instability and an increased risk of falls [37]. As balance and postural control are key components of frailty, this mechanistic pathway offers a plausible explanation for the observed relationship between occlusal status and physical decline. Furthermore, impaired mastication affects dietary choices and reduces the enjoyment of eating, often leading to inadequate nutritional intake. Malnutrition is a well‐recognized risk factor for frailty, and previous studies have established strong interconnections among masticatory function, nutritional status, and frailty [7, 30, 38, 39, 40]. Although insufficient nutritional intake may mediate this association, recent findings suggest that poor chewing and frailty are independently linked to nutritional status [30], highlighting the complexity of this relationship. Collectively, these findings show that reduced posterior occlusal support is linked to frailty risk. Further studies should clarify causal pathways and test whether interventions to improve occlusal function reduce frailty.

The results of this study, which showed that the association between the Eichner classification and frailty remained significant even after adjusting for the tooth number, also confirmed that the impact of occlusal contact loss was not fully captured by the tooth number alone. These findings align with previous research where univariable analyses indicated that both chewing ability and tooth number were significantly associated with frailty; however, when analyzed together, only chewing ability retained its significant association [8]. Notably, the number of teeth does not fully reflect oral function because an unfavorable tooth arrangement without occlusal contact can compromise mastication. Given that occlusal contact plays a crucial role in determining masticatory efficiency, it may serve as a more accurate indicator of dental status and its impact on systemic health [41]. Our findings suggest that the effect of tooth loss on frailty is primarily mediated by the presence or absence of functional occlusion, particularly in the molar region. Future longitudinal studies are needed to clarify the causal mechanisms underlying this relationship and develop targeted interventions that support occlusal function as a strategy for frailty prevention.

This study had some limitations that must be acknowledged. First, the CFI calculation in this study deviated from the original methodology owing to the differences in treatment codes between Japan and the United States. Certain codes were excluded from the CFI calculation, potentially leading to misclassifications. Nevertheless, in accordance with previous research, we adjusted the weights of the other codes to ensure consistency with the original CFI scoring system [20]. While the CFI is useful for population‐level research with administrative databases, its sensitivity and specificity are lower than those of clinically validated measures such as the Fried and the Kihon Checklist. This limitation should be considered when interpreting our results. Previous Japanese studies using validated frailty measures have reported that the proportions of robust, pre‐frail, and frail individuals range from 26% to 56%, 28% to 65%, and 4.6% to 39%, respectively [42]. In our study, which included a younger population (mean age, approximately 50 years), the corresponding proportions were 60%, 35%, and 5%, respectively. However, when analyzed by age group, frailty prevalence exceeded 10% among individuals aged ≥ 65 years (Figure S1), aligning with previously reported rates for populations of similar age [20, 42]. Second, the JMDC database primarily consists of Japan's working‐age population and its dependents, with limited representation of individuals aged > 75 years. This age restriction limited our ability to fully investigate the impact of posterior occlusal support loss on frailty in older adults. Given that the database focuses on younger populations, frailty events are relatively infrequent, which may limit the generalizability of our findings to older age groups. Third, while we adjusted for conventional confounders available in the database, residual confounding factors such as social participation may still influence the observed association between posterior occlusal support loss and frailty. Frailty is a multifaceted condition encompassing physical, social, and psychological factors, and masticatory difficulty may exacerbate physical frailty by limiting social engagement. Importantly, key covariates such as socioeconomic status, educational attainment, and nutritional status were not available in the JMDC database, and their absence should be taken into account when interpreting the results. However, the E‐values suggest that residual confounding was unlikely to affect the observed associations (Table S8). Fourth, the presence and severity of periodontitis were not considered despite evidence suggesting that inflammatory processes and immune responses contribute to frailty [43]. Direct data on periodontal status were unavailable in the claims dataset. The number of remaining teeth can serve as a proxy for past periodontitis, as individuals with fewer teeth are more likely to have experienced severe disease. However, this measure does not capture the current periodontal status, and residual confounding remains possible. In addition, while our analysis accounted for the use of validated dentures [44], the claims database lacked data on implant‐supported prostheses, which we regard as a study limitation. Fifth, the exposure (Eichner's classification) was measured only at baseline and was not updated over time. Consequently, the models do not capture individual‐level frailty onset or progression, and causal inference regarding frailty development remains limited.

## 5 Conclusion

Using a nationwide longitudinal dataset, we observed that individuals with reduced occlusal support, as classified using the Eichner classification, exhibited a higher risk of frailty based on CFI assessment. These results suggest that posterior occlusal contact loss is a potential oral health risk factor for frailty. Given the aging population, further studies are warranted to examine the causal mechanisms underlying the relationship between occlusal support and frailty, and to evaluate whether interventions targeting occlusal function could potentially modify frailty risk.

## Author Contributions

All authors satisfy the authorship criteria outlined in the Uniform Requirements for Manuscripts Submitted to Biomedical Journals. T.M., Y.T., T.K., K.O., and K.T. contributed to the study's concept and design. K.T. carried out data acquisition, while T.M., Y.T., T.K., K.O., and K.T. were responsible for analyzing and interpreting the data. T.M. prepared the initial draft of the manuscript, with Y.T., T.K., K.O., and K.T. providing critical revisions to enhance the manuscript's intellectual content.

## Funding

This study was funded by Grants‐in‐Aid for Scientific Research (22H03299, 23K24557) from the Japan Society for the Promotion of Science (JSPS) KAKENHI and Health and Labor Sciences Research Grants (23FA1022) from the Ministry of Health, Labor, and Welfare.

## Ethics Statement

This study used anonymized data sourced from the JMDC Claims Database, a commercially available resource, in compliance with Japan's Act on the Protection of Personal Information. Anonymization followed the standards outlined in the Next‐Generation Medical Infrastructure Act. In line with Japan's ethical guidelines for clinical research, studies that use anonymized information do not require approval from ethics review boards. This study adhered to the principles of the Declaration of Helsinki.

## Consent

As the data were anonymized, informed consent was not required for their collection or use.

## Conflicts of Interest

The authors declare no conflicts of interest.

## Supporting information


**Data S1:** Supporting Information.

## Data Availability

The data that support the findings of this study are available on request from the corresponding author. The data are not publicly available due to privacy or ethical restrictions.
