# Supplementary material for: Role of Posterior Occlusal Support in the Development of Frailty Among Japanese Adults: A Longitudinal Cohort Study
Source: Geriatr Gerontol Int. 2026 Feb 8;26(2):e70405. doi: 10.1111/ggi.70405 (PMC12883313; doi:10.1111/ggi.70405)
Supplement: Supplementary file 1 — Data S1: Supporting Information. [file GGI-26-0-s001.docx]

**Supplementary information**

**Supplementary Table S1. Weights for each item in the claims-based frailty Index (CFI).**

**Supplementary Table S2. Definition of used variables.**

**Supplementary Table S3. Interaction between time points and patterns of Eichner classification in relation to changes in frailty.**

**Supplementary Table S4. Univariable and multivariate GEE model for frailty (excluding participants with missing data).**

**Supplementary Table S5. Univariable and multivariate GEE model for frailty (continuous data).**

**Supplementary Table S6. Interaction between sex and patterns of Eichner classification in relation to changes in frailty.**

**Supplementary Table S7. Multivariate GEE model for frailty (sex stratified).**

**Supplementary Table S8. Robustness to unmeasured confounding (E-values) of the association between Eichner classification and frailty.**

**Supplementary Figure 1. Prevalence of frailty across age categories.**

**Supplementary Table S1. Weights for each item in the claims-based frailty Index (CFI).**

| **CFI item** | **Weight** |  | **CFI item** | **Weight** |  | **CFI item** | **Weight** |
| --- | --- | --- | --- | --- | --- | --- | --- |
| 1 | 0.010249 |  | 19 | -0.00107 |  | 37 | 0.024795 |
| 2 | 0.020921 |  | 20 | 0.030642 |  | 38 | 0.017329 |
| 3 | 0.014634 |  | 21 | 0.033812 |  | 39 | 0.00037 |
| 4 | 0.014035 |  | 22 | 0.035027 |  | 40 | 0.00685 |
| 5 | -0.00903 |  | 23 | 0.027331 |  | 41 | -0.01472 |
| 6 | -0.00107 |  | 24 | 0.001867 |  | 42 | 0.001286 |
| 7 | 0.007731 |  | 25 | 0.021731 |  | 43 | 0.010443 |
| 8 | 0.018984 |  | 26 | -0.00916 |  | 44 | -0.00407 |
| 9 | -0.00993 |  | 27 | 0.020868 |  | 45 | 0.019706 |
| 10 | 0.014969 |  | 28 | 0.022805 |  | 46 | 0.03596 |
| 11 | -0.01416 |  | 29 | 0.004596 |  | 47 | 0.018913 |
| 12 | -0.01197 |  | 30 | 0.000881 |  | 48 | -0.0025 |
| 13 | 0.083561 |  | 31 | 0.006833 |  | 49 | -0.00333 |
| 14 | 0.037193 |  | 32 | 0.018438 |  | 50 | 0.003364 |
| 15 | 0.023827 |  | 33 | 0.018702 |  | 51 | 0.002078 |
| 16 | 0.070987 |  | 34 | -0.03753 |  | 52 | -0.02127 |
| 17 | 0.00722 |  | 35 | 0.009879 |  |  |  |
| 18 | 0.005952 |  | 36 | -0.00093 |  |  |  |

**Supplementary Table S2. Definition of used variables.**

| Claims-based frailty Index (CFI) | |
| --- | --- |
| Data source | - The medical claims data |
| Definition | - The CFI was defined using the 52 items based on the ICD-10 code presented by Nakatsuka et al. - The record with a suspicious flag was excluded. - The initial appearance of diseases record was used. |
| The number of teeth | |
| Data source | - Dental records of the dental claims data |
| Definition | - The dental record data of those who took periodontal examination was used. - The following dental procedure code was considered as periodontal examination: 304000410, 304000510, 304000610, 304000710, 304000810, 304000910. - Deciduous teeth, supernumerary teeth, and third molar were excluded. - The tooth condition of missing, dental implants, and residual root were considered as missing teeth. |
| Denture use | |
| Data source | - Dental records of the dental claims data |
| Definition | - The following dental procedure code was considered as denture use: 308002510, 308002610, 308002710, or 308004210. |
| Age, sex | |
| Data source | - The ledger of the beneficiary |
| Definition | - Age was calculated based on the month of birth and the inclusion date. - Sex and types of insured person variables were also available in the beneficiary's ledger. |
| Charlson’s comorbidity index | |
| Data source | - The medical claims data |
| Definition | - Charlson comorbidity index updated by Quan et al. - We collected the disease names which were recorded a year before the inclusion date. - The disease name was collected based on the ICD-10 code presented by Glasheen et al. |
| Depression | |
| Data source | - The medical claims data |
| Definition | - The following ICD-10 code was considered as depression: F32 and F33. - The record with a suspicious flag was excluded. |
| Dementia | |
| Data source | - The medical claims data |
| Definition | - The following ICD-10 code was considered as dementia: F00–F03 and G30–G31, excluding G319. - The record with a suspicious flag was excluded. |
| Body mass index (BMI) | |
| Data source | - The health checkup data |
| Definition | - The cut off was ≥25.0/18.5–25.0/<18.5 kg/m^2^ |
| Smoking | |
| Data source | - The health checkup data (questionnaire) |
| Definition | - Question: “Do you smoke currently?” - Answer: “Yes,” “No” |
| Alcohol consumption | |
| Data source | - The health checkup data (questionnaire) |
| Definition | - Question: “Frequency of drinking alcohol (Japanese sake, Shochu, Beer, Wine, etc.)” - Answer: “1. Every day,” “2. Occasionally,” “3. Seldom/Never.” - Coding: “1” OR “2” → “Yes”; “3” → “No” |
| Physical activity | |
| Data source | - The health checkup data (questionnaire) |
| Definition | - Question: “Engage in walking or equivalent physical activity for at least one hour per day in daily life.” - Answer: “Yes,” “No” |

[Ref.]

- Nakatsuka K, Ono R, Murata S, Akisue T, Fukuda H. Claims-based Frailty Index in Japanese Older Adults: A Cohort Study Using LIFE Study Data. J Epidemiol. 2024;34(3):112-118. doi:10.2188/jea.JE20220310
- Quan H, Li B, Couris CM, Fushimi K, Graham P, Hider P, Januel J-M, Sundararajan V. Updating and validating the charlson comorbidity index and score for risk adjustment in hospital discharge abstracts using data from 6 countries. American Journal of Epidemiology. 2011;173(6):676-682. doi:10.1093/aje/kwq433
- Glasheen WP, Cordier T, Gumpina R, Haugh G, Davis J, Renda A. Charlson comorbidity index: Icd-9 update and icd-10 translation. American health & drug benefits. 2019; 12(4):188.

**Supplementary Table S3. Interaction between time points and patterns of Eichner classification in relation to changes in frailty.**

|  | **Overall** | | |
| --- | --- | --- | --- |
|  | **OR** | **95% CI** | **P-value** |
| Interaction effect: Eichner classification | | | |
| Eichner B # time points | 1.00 | 0.98–1.02 | 0.75 |
| Eichner C # time points | 0.98 | 0.94–1.01 | 0.16 |

The generalized estimating equation (GEE) models were used to estimate the odds ratios (ORs) for frailty changes, with Eichner classification and time point as the main variables of interest. Eichner A: Posterior occlusal support in all quadrants, Eichner B: Partial loss of posterior occlusal support, Eichner C: No posterior occlusal support. OR represents odds ratio, and CI represents confidence interval.

**Supplementary Table S4. Univariable and multivariate GEE model for frailty (excluding participants with missing data).**

|  | **Univariable** | | |  | **Multivariable** | | |
| --- | --- | --- | --- | --- | --- | --- | --- |
|  | **OR** | **95% CI** | **P-value** |  | **OR** | **95% CI** | **P-value** |
| Eichner classification (reference: Eichner_A) | | | | | | | |
| Eichner_B | 2.03 | 1.94–2.11 | < 0.001 |  | 1.20 | 1.11–1.29 | < 0.001 |
| Eichner_C | 2.45 | 2.28-2.63 | < 0.001 |  | 1.39 | 1.20–1.61 | < 0.001 |

The generalized estimating equation (GEE) models assess frailty as the outcome, with the Eichner classification as the explanatory variable. Eichner A: Posterior occlusal support in all quadrants, Eichner B: Partial loss of posterior occlusal support, Eichner C: No posterior occlusal support. OR represents odds ratio, and CI represents confidence interval.

**Supplementary Table S5. Univariable and multivariate GEE model for frailty (continuous data).**

|  | **Univariable** | | |  | **Multivariable** | | |
| --- | --- | --- | --- | --- | --- | --- | --- |
|  | **OR** | **95% CI** | **P-value** |  | **OR** | **95% CI** | **P-value** |
| Eichner classification (reference: Eichner_A) | | | | | | | |
| Eichner_B | 2.03 | 1.94–2.11 | < 0.001 |  | 1.11 | 1.02–1.20 | 0.011 |
| Eichner_C | 2.45 | 2.28-2.63 | < 0.001 |  | 1.27 | 1.05–1.54 | 0.015 |

The generalized estimating equation (GEE) models evaluate frailty as the outcome, with the Eichner classification as the explanatory variable. Eichner A: Posterior occlusal support in all quadrants, Eichner B: Partial loss of posterior occlusal support, Eichner C: No posterior occlusal support. OR represents odds ratio, and CI represents confidence interval.

**Supplementary Table S6. Interaction between sex and patterns of Eichner classification in relation to changes in frailty.**

|  | **Overall** | | |
| --- | --- | --- | --- |
|  | **OR** | **95% CI** | **P-value** |
| Interaction effect: Eichner classification | | | |
| Eichner B # Women | 1.23 | 1.13–1.35 | < 0.001 |
| Eichner C # Women | 1.45 | 1.24–1.70 | < 0.001 |

The generalized estimating equation (GEE) models were used to estimate the odds ratios (ORs) for frailty changes, with Eichner classification and sex as the main variables of interest. Eichner A: Posterior occlusal support in all quadrants, Eichner B: Partial loss of posterior occlusal support, Eichner C: No posterior occlusal support. OR represents odds ratio, and CI represents confidence interval.

**Supplementary Table S7. Multivariate GEE model for frailty (sex stratified).**

|  | **Men (n = 230,308)** | | |  | **Women (n = 155,962)** | | |
| --- | --- | --- | --- | --- | --- | --- | --- |
|  | **OR** | **95% CI** | **P-value** |  | **OR** | **95% CI** | **P-value** |
| Eichner classification (reference: Eichner_A) | | | | | | | |
| Eichner_B | 1.18 | 1.08–1.28 | < 0.001 |  | 1.28 | 1.14–1.44 | < 0.001 |
| Eichner_C | 1.31 | 1.11–1.55 | 0.001 |  | 1.57 | 1.24–1.99 | < 0.001 |

The generalized estimating equation (GEE) models evaluate frailty as the outcome, with the Eichner classification as the explanatory variable. Eichner A: Posterior occlusal support in all quadrants, Eichner B: Partial loss of posterior occlusal support, Eichner C: No posterior occlusal support. OR represents odds ratio, and CI represents confidence interval.

**Supplementary Table S8. Robustness to unmeasured confounding (E-values) of the association between Eichner classification and frailty.**

|  | Multivariable | |
| --- | --- | --- |
|  | E-value for point estimate | E-value for confidence limit |
| Eichner classification (reference: Eichner A) | | |
| Eichner B | 1.71 | 1.51 |
| Eichner C | 2.13 | 1.71 |

Eichner A: Posterior occlusal support in all quadrants, Eichner B: Partial loss of posterior occlusal support, Eichner C: No posterior occlusal support.


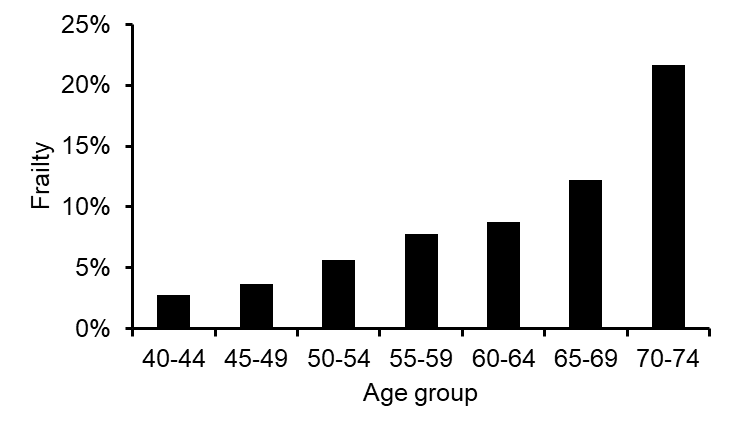


**Supplementary Figure 1. Prevalence of frailty across age categories.**

Prevalence of frailty by 5-year age group in individuals aged 40 to 74 years.
